# Readiness, Availability and Utilization of Rural Vietnamese Health Facilities for Community Based Primary Care of Non-communicable Diseases: A CrossSectional Survey of 3 Provinces in Northern Vietnam

**DOI:** 10.15171/ijhpm.2018.104

**Published:** 2018-11-04

**Authors:** David B. Duong, Hoang Van Minh, Long H. Ngo, Andrew L. Ellner

**Affiliations:** ^1^Department of Medicine, Brigham and Women’s Hospital, Center for Primary Care, Harvard Medical School, Boston, MA, USA.; ^2^Hanoi University of Public Health, Hanoi, Vietnam.; ^3^Department of Medicine, Beth Israel Deaconess Medical Center, Harvard Medical School, Boston, MA, USA.; ^4^Division of Global Health Equity, Brigham and Women’s Hospital, Center for Primary Care, Harvard Medical School, Boston, MA, USA.

**Keywords:** Non-communicable Diseases, Primary Care, Primary Healthcare, Disadvantaged Populations, Vietnam

## Abstract

**Background:** Vietnam’s network of commune health centers (CHCs) have historically managed acute infectious diseases and implemented national disease-specific vertical programs. Vietnam has undergone an epidemiological transition towards non-communicable diseases (NCDs). Limited data exist on Vietnamese CHC capacity to prevent, diagnose, and treat NCDs. In this paper, we assess NCD service readiness, availability, and utilization at rural CHCs in 3 provinces in northern Vietnam.

**Methods:** Between January 2014 and April 2014, we conducted a cross-sectional survey of a representative sample of 89 rural CHCs from 3 provinces. Our study outcomes included service readiness, availability of equipment and medications, and utilization for five NCD conditions: hypertension, diabetes, chronic pulmonary diseases, cancer, and mental illnesses.

**Results:** NCD service availability was limited, except for mental health. Only 25% of CHCs indicated that they conducted activities focused on NCD prevention. Patient utilization of CHCs was approximately 223 visits per month or 8 visits per day. We found a statistically significant difference (P<.05) for NCD service availability, medication availability and CHC utilization among the 3 provinces studied.

**Conclusion:** This is the first multi-site study on NCD service availability in Vietnam and the first study in a mountainous region consisting predominately of ethnic minorities. Despite strong government support for NCD prevention and control, Vietnam’s current network of CHCs has limited NCD service capacity.

## Background

Worldwide, non-communicable diseases (NCDs) are responsible for over 38 million deaths each year, with 28 million annual deaths occurring in low- and middle-income countries (LMICs).^1^ The World Health Organization’s (WHO’s) definition of NCDs include diabetes, cancer, cardiovascular diseases (CVDs), chronic lung diseases (CLDs) and mental health.^1^ Coinciding with socio-economic reforms that transformed Vietnam from one of the world’s poorest countries (1991 per capita income of US$137) to an LMIC (2016 per capita income of US$2170), Vietnam has experienced a shift in disease burden from communicable to NCD.^2,3^ The poverty headcount has fallen from 58% in the early 1990s to under 10% by 2010 and the country has achieved almost all the health-related millennium development goals.^4,5^

From 1986 to 2008, the proportion of all hospital admissions attributable to NCDs increased from 39% to 69%, and NCD deaths rose from 42% to 63%.^6^ Coincident with this shift, there is increasing over-utilization of provincial and central level hospitals and under-utilization of commune health centers (CHCs) – a politically and socially volatile problem for the government that will be exacerbated as more people gain access to healthcare through the country’s effort to achieve universal healthcare (UHC).^7,8^

There is strong evidence that primary care can deliver better health outcomes at lower cost.^9-11^ People with NCDs and those with NCD risk factors require long-term care that is proactive, patient-centered, community-based and sustainable.^11,12^ However, there are limited data from LMICs on NCD service readiness, availability and utilization in primary care facilities.^11-13^ Without such data, it is difficult for governments and international organizations to develop appropriate interventions, and to assess the quality and impact of the services provided.^12,13^ The absence of necessary data perpetuates an inadequate strategy and response at the primary care level.^11,12^

### Vietnamese Healthcare System

The Vietnamese healthcare system retains a hierarchical structure comprised of CHCs, district health centers (DHCs) and provincial and national referral hospitals. CHCs are intended to be the entry-point for the health system. Each CHC serves a population of 5000-10 000 people. The DHC is the first level of the Healthcare system providing in-patient hospital services. Provincial hospitals provide tertiary care and national hospitals are quaternary referral centers. CHCs have traditionally been responsible for implementing national target programs (NTPs) in the community through funding from the central government. NTPs are single interventions (such as immunizations) or condition-specific (such as malnutrition or tuberculosis). CHCs also provide examination and treatment for common complaints (eg, upper respiratory infections, diarrheal disease, superficial wound closure), prenatal and postnatal care, spontaneous-uncomplicated vaginal deliveries, and provide referrals to the DHC.

### Non-communicable Disease Response in Vietnam

Vietnam was the first country in Southeast Asia to establish a National Program on NCD Prevention and Control in 2002.^14^ From this National Program, Vietnam’s Ministry of Health (MoH) created 4 NTPs to address mental health, hypertension, cancer and diabetes.^15-18^ An independent review of the 4 NTPs in 2011 concluded that despite the political will for NCD prevention and control, the NTPs resulted in limited population health gains.^19^ The review identified 2 major limitations: (1) the NTPs were implemented as individual disease programs focused on treatment rather than prevention, and (2) the NTPs were centrally funded without incorporation into social health insurance or local financing, leading to long-term unsustainability.^19^ Additional studies provided further support for this assessment. A study of 18 CHCs in one district concluded that the CHCs were inadequate to provide the NCD-related health needs of the population.^20^ Another study found that the proportion of people with NCDs who used at least one outpatient service and at least one inpatient health service during the last 12 months were 68.1% and 10.7% respectively,^21^ with lower utilization among ethnic minorities and people without health insurance.^22,23^ Health worker knowledge to address NCDs was also found to be insufficient.^24^

Aside from the studies described above, the data around NCD service availability, readiness and utilization in Vietnam are limited. The majority of published literature consists of descriptive, cross-sectional studies on the prevalence of NCD risk factors and/or diseases, with a focus on major urban areas.^25-29^ Within the last ten years, the WHO has supported time-limited (less than 1-year) pilots on hypertension diagnosis and management at select CHCs, and the Pfizer and Novartis Foundations have supported 2 programs focused on screening for and managing hypertension and diabetes at public and private CHCs in Ho Chi Minh City. No data are published on the results of these implementation efforts.

The objective of this study was to assess NCD service availability, readiness and utilization for prevention, screening, and treatment services across representative CHCs from rural provinces in northern Vietnam. This objective differs from previous studies which focused on a single district within one province. We hypothesized that NCD service readiness, availability and utilization would be limited and that there would be less capacity for managing NCDs in provinces with less wealth and a higher representation of ethnic minorities.

## Methods

### Study Setting

Between January 2014 and April 2014, we conducted a cross-sectional survey of public CHCs in 3 provinces in northern Vietnam, representing 3 different geo-socio-economic-ethnographic regions: the Red River delta (Hai Phong), the northern midlands (Bac Giang), and the mountainous area (Dien Bien) (Figure).

**Figure:**
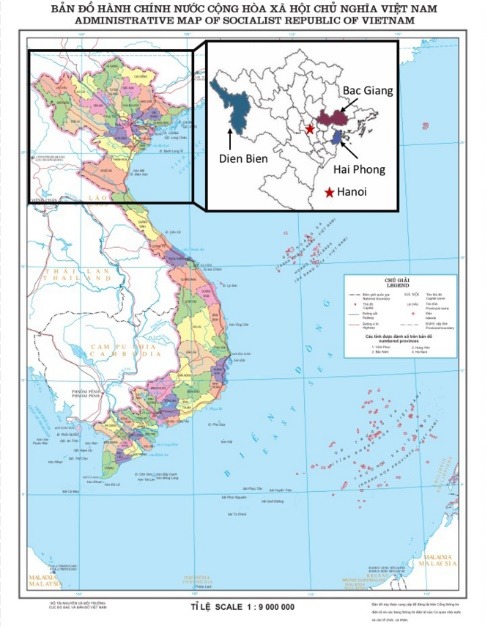
Geographical Location of Study Provinces.
Map Source: Vietnam Department of Survey, Mapping and Geographical Information. Center of Survey and Mapping Data (http://bandovn.vn/(X(1)S(bhqqpxu44d0apoubrs2anuyu))/vi/page/mau-ban-do-hanh-chinh-nuoccong-hoa-xa-hoi-chu-nghia-viet-nam-181?AspxAutoDetectCookieSupport=1). Accessed August 1, 2018.

Hai Phong has a robust coal mining industry with an annual GDP of $1819/year, and a population consisting mainly of ethnic Vietnamese (Kinh). Bac Giang has an industrial manufacturing base with an annual per capita GDP of $1095/year and a population consisting of mainly Kinh and 12.4% ethnic minorities. Dien Bien has an economy dependent on subsistence farming with an annual per capita GDP of $809/year and a population that is 80% ethnic minorities (Table 1). Dien Bien has the highest infant and maternal mortality and percentage of under-5 malnutrition. Dien Bien also has the highest percentage of current smokers and the highest rate of alcohol consumption. No data currently exist for NCD prevalence by province in Vietnam.

**Table 1:** Study Province Characteristics

**Province**	**Hai Phong (Red River Delta, n = 20)**	**Bac Giang (Northern Midlands, n = 37)**	**Dien Bien (Mountainous, n = 32)**
**General Data (2012)** ^a^			
Annual GDP per capita (VND(mil))	38	23	17
Literacy rate (%)	98.5	97.7	71.4
Area (km^2^)	1527.4	3849.7	9562.9
Population (1000)	1925.2	1593.2	527.3
Physicians (per 1000 people)	1.4	1.1	0.4
Physician Assistants (per 1000 people)	0.7	1.4	1.1
Nurses (per 1000 people)	2.7	1.5	0.5
Midwives (per 1000 people)	0.5	0.3	0.2
Pharmacists (per 1000 people)	0.3	0.4	0.2
Number of hospitals	24	16	14
Number of health facilities	224	230	112
**Disease Epidemiology (2014)** ^b^			
Prevalence of HIV per 100 000 people (2015) (%)^c^	0.38	0.10	0.82
Infant mortality (per 1000 live births)	12.2	15.3	34.4
Under-5 moderate malnutrition (%)	8.0	15.2	19.2
Maternal mortality (total deaths)^d^	0	2	10
Malaria (cases per 100 000 people)	3.2	5.9	54.9
**Risk Factors for NCD (2010)** ^e^			
Tobacco (% current smokers)	16	20	25
Alcohol intake (% 1-4 days/week)	7	11	16

Abbreviations: GDP, Gross domestic product; NCD, non-communicable disease. ^a^Government of Vietnam. General Government Statistics Office; Government of Vietnam; Hanoi: 2012. http://www.gso.gov.vn/default_en.aspx?tabid=783 (accessed May 1, 2014). ^b^Vietnam Ministry of Health. Heath Statistics Year Book 2014. Medical Publishing House; Hanoi: 2014. ^c^Vietnam Administration for HIV/AIDS Control. Vietnam Administration for HIV/AIDS Control Annual Report 2015. Vietnam Administration for HIV/AIDS Control; Hanoi: 2015. ^d^Total number of deaths from hemorrhage, eclampsia, tetanus, uterine rupture and infection. ^e^Vietnam National Institute of Nutrition. Vietnam National Institute of Nutrition Country Survey—2012. Vietnam National Institute of Nutrition; Hanoi: 2012.

### Health Facility selection

We selected one province from each of the 3 geographical regions (total of 24 provinces). Three provinces (Phu Tho, Thai Nguyen, Thai Binh) had previous NCD interventions and were not considered. We separated the 21 remaining provinces into their geographical regions and selected the province in each region that had the highest-weighted composite score of prevalence of self-reported alcohol and tobacco consumption—risk factors that are positively associated with NCDs.^1,27,30^ We extracted data on these indicators from the Vietnam National Nutrition Survey.^31^ We selected 2 districts, one peri-urban and one rural, in each province based on convenience sampling, and included all CHCs within each district to receive mail-in questionnaires (N = 89). We chose 3 CHCs per district for visits by the research team for in-depth surveying and checking the questionnaires for accuracy (n = 18). We chose those CHCs based on (1) being representative of the economic and geographical diversity in the district, (2) travel time from the DHC, and (3) convenience of travel to the CHC.

### Power Analysis

At the design phase of the study, we estimated the NCD service availability prevalence to be under 50% for the 5 conditions (diabetes, CVDs, CLDs, cancer, mental illness) and aimed to have a sufficiently large sample size of provinces to achieve a precision of less than 15% of the estimate. We focused on CVDs and set the prevalence for CVD service availability at 40% with a standard error of 6% or less (from 15% of 40%; thus from 36%-46%). We used the formula for standard error of a probability sqrt(P*(1-P)/N) where P is the prevalence (set at 40%). Setting this equation equal to 6%, and solving for the desired sample size N, we computed N = 67. We collected data from 89 CHCs, exceeding the 15% threshold (ie, lower than 15%).

### Data Collection

We collected data on CVDs (hypertension, myocardial infarction, stroke), diabetes, CLDs (chronic obstructive lung disease, asthma), mental illness (schizophrenia, epilepsy) and cancer. We chose this group of diseases because they are included in both the WHO definition of NCDs and targeted by the Vietnam NTP.^11,14-19^

We collected data using a modified version of the WHO Service Availability and Readiness Assessment (SARA) questionnaire, which allowed for a systematic way to assess our 3 study variables (NCD service readiness, availability and utilization) through a single tool.^32^

We measured service readiness by the availability of NCD related medication and equipment (cross-referenced between SARA and the 2012 Vietnam national essential medications and equipment list for CHC, and included only if they were on both lists). We measured service availability through the types of NCD services available (ie, prevention, screening, treatment, management). We measured service utilization by asking the number of patients served by the CHC the previous day and the previous month for all causes and for the 5 NCDs.^32,33^

We developed the final version of the modified SARA tool in English. The tool was translated into Vietnamese by a certified translator and back-translated into English to ensure accuracy. We piloted the tool at a non-study site and revised the tool based on that experience. We created 2 versions of the survey instrument to be administered by mail and in person. All 3 members of the research team were involved in the development of the modified SARA tool and participated in a half-day training on the study tools. We sent the modified SARA tool to the provincial department of health who distributed the tool to the 6 DHCs. The DHCs distributed the tool to all CHCs in their respective districts, and all CHC directors received an in-person tutorial on completing the tool. Two weeks after the receiving the SARA tool, CHC directors returned the completed tool. The 3-member research team visited all DHCs to collect and audit the completed surveys, and visited 3 CHCs per district to check survey accuracy (n = 18).

### Data Analysis

We collected data on paper questionnaire forms and entered it into an electronic database. We used Stata for analysis. We examined frequency distributions of all analysis variables to check for data that were outside of the expected range and known categories. We categorized variables for the availability of NCD medications and equipment into dichotomous variables for analysis. We reported mean and standard deviation (SD) for continuous variables and percentages for categorical variables. For comparison of NCD prevalence estimates among the 3 provinces regarding service readiness, availability and utilization, we reported the chi-square or Fisher exact test statistic *P* values. If at least 25% of the cells in the table had the expected cell frequency less than 5, we then used Fisher’s exact test. For service readiness and availability, *P* values are from chi-square or Fisher’s exact test testing the null hypothesis that the population proportions are equal among the 3 provinces. For clinic utilization, *P* values are from analysis of variance (ANOVA) (if the distribution is nearly normal) or Kruskal-Wallis test (if the distribution deviates from normality) testing the null hypothesis that the population means of the number of visits were equal among the 3 provinces. Type-I error was set at 0.05. We also reported the 95% CI of these estimates.

The institutional review boards of Hanoi Medical University and Harvard Medical School approved this study. Healthcare workers’ data were collected without any identifying information after written consent was obtained.

## Results

We surveyed 89 CHCs from the following geographical regions: Hai Phong (Red River delta) (n = 20), Bac Giang (northern midlands) (n = 37) and Dien Bien (mountainous) (n = 32). There was a 100% response rate among CHCs.

### Non-communicable Disease Service Availability

CHCs in all 3 regions reported providing NCD services (screening and/or treatment) for the 5 major NCD groups. We found a statistically significant difference (*P* = .000) between the geographical regions for service availability for diabetes, CVDs, and CLDs (Table 2).

**Table 2:** NCD Service Availability (N = 89)

	**Province Total**	**Hai Phong (Red River Delta, n = 20)**	**Bac Giang (Northern Midlands, n = 37)**	**Dien Bien (Mountainous, n = 32)**	***P*** **Value** ^b^
Diabetes	53	85	68	22	<.0001
CVD^a^	64	90	78	38	<.0001
CLD^a^	39	35	80	5.5	<.0001
Cancer	22	35	28	11	.082
Mental Illness^a^	96	95	96	97	.903

Abbreviations: NCD, non-communicable disease; CVD; cardiovascular disease; CLD, chronic lung diseases. ^a^CVD is hypertension, myocardial infarction, stroke; CLD is chronic obstructive lung disease (COPD) and asthma; Mental health: epilepsy, schizophrenia. ^b^*P* values are from chi-square tests testing the null hypothesis that the population proportions are equal among the 3 provinces. All numbers in the table are percentages. All variables here are binary (yes/no).

Of the CHCs providing diabetes (n = 47) and cancer (n = 19) services, screening was the predominant service available (78%, diabetes; 52%, cancer), compared to treatment (19%, diabetes; 19%, cancer). Cancer treatment consists only of morphine for palliative care. In contrast, services for screening and treatment of hypertension and CLD services were comparable. Fifty-seven CHCs reported delivering hypertension services. Of these, 68% offered screening (risk stratification and blood pressure measurement) and 61% offered treatment. Only 36 CHCs provided services for CLDs. Among these CHCs, more provided treatment (86%) than screening (72%). For schizophrenia and epilepsy, 83 out of the total 89 CHC surveyed provided services for both screening (81%) and treatment (92%).

### Non-communicable Disease Preventative Service Availability

Less than 25% of CHCs conducted NCD prevention programs focused on alcohol use, tobacco use, inactivity and unhealthy diet (Table 3). The Red River delta and northern midlands region offered more preventative services than the mountainous region for prevention services around inactivity (*P* = .006), diet (*P* = .035), and tobacco use (*P* = .000) (Table 3). Preventative services consisted of mass communication campaigns via health information broadcast through loudspeakers rather than individualized patient counselling.

**Table 3:** NCD Preventative Service

	**Province Total**	**Hai Phong (Red River Delta, n = 20)**	**Bac Giang (Northern Midlands, n = 37)**	**Dien Bien (Mountainous, n = 32)**	***P*** **Value** ^a^
Alcohol	22	11	34	17	.092
Inactivity	20	21	37	6	.006
Diet	20	21	34	9	.035
Tobacco	25	5	59	6	<.0001

Abbreviation: NCD, non-communicable disease. ^a^*P* values are from chi-square tests testing the null hypothesis that the population proportions are equal among the 3 provinces. All numbers in the table are percentages. All variables here are binary (yes/no).

### Non-communicable Disease Service Readiness

In all NCD disease groups, except mental health, equipment availability is higher than medication availability. The mountainous region had the lowest availability of CVD medications as compared to the other regions (*P* = .005). There was no difference in the availability of NCD diagnostic equipment between the regions.

Ninety-eight percent of CHCs (n = 88) had at least one type of NCD equipment. In general, availability of equipment for diabetes (95%) and hypertension (96%) was higher, compared to equipment for CLDs (76%). In contrast, only 47% of CHCs had at least one type of first-line NCD medication. Only 3% of CHCs carried at least one type of diabetes medications (metformin, glibenclamide or insulin). 9% of CHCs had available salbutamol and/or beclomethasone inhalers for the treatment of CLDs. 31% of CHC had at least one type of anti-hypertensive (beta-blocker, calcium channel blockers, angiotensin inhibitors, diuretic) or aspirin.

### Clinic Utilization

The mean number of outpatient visits to CHCs for all causes was 223 patients per month, or approximately 7 to 8 patients per day. The variation between geographic regions for patient volume is significant (*P* = .000): the Red River delta had the highest mean number of patient visits per month (317) and the previous day (23); the mountainous had the least mean number of patient visits per month (166) and the previous day (8). For NCD specific patient visits over the past month, the mean number of patients per month for CVD is 10, diabetes is 1.5 (SD = 3.4), CLD is 4 (SD = 9.4), cancer is 0.5 (SD = 1.3), and mental health is 15 (SD = 15.9). In aggregate, NCD visits make up less than 15% of all visits to CHCs. The difference between regions for NCD visits also significantly varies for diabetes (*P* = .004), CLD (*P* = .000), cancer (*P* = .000) and mental illness visits (*P* = .000). The mountainous region has the lowest mean number of patient visits in every NCD category except for mental illness (*P* = .000) (Table 4). Table 4 demonstrates a statistically significant difference of the mean volume of patient visits among the 3 provinces and the overall mean volume of patient visits, except for CVDs.

**Table 4:** Mean Patient Monthly Volume

	**Province Total**	**Hai Phong (Red River Delta, n = 20)**	**Bac Giang (Northern Midlands, n = 37)**	**Dien Bien (Mountainous, n = 32)**	***P*** **Value** ^b^
Total outpatient vis-its (mon)	223.5	317.2	233.7	166.6	<.0001
Total outpatient vis-its (yesterday)	15.1	23.3	18.2	8.2	<.0001
DM visits/month	1.4	1.2	2.3	0.9	.004
CVD^a^ visits/month	10.7	13.4	12.5	7.8	0.185
CLD^a^ visits/month	4.0	0.3	8.4	1.8	<.0001
Cancer visits/month	0.5	0.2	1.1	0.1	<.0001
Mental health^a^ visits/month	15.1	2.3	12.6	23.1	<.0001

Abbreviations: NCD, non-communicable disease; DM, diabetes mellitus; CVD, cardiovascular disease; CLD, Chronic lung diseases. ^a^CVD is hypertension, myocardial infarction, stroke; CLD is chronic obstructive lung disease (COPD) and asthma; Mental health: epilepsy, schizophrenia. ^b^*P* values are from ANOVA or Kruskal-Wallis tests testing the null hypothesis that the population means of the number of visits are equal among the 3 provinces. All numbers in the table are mean. All variables here are continuous.

## Discussion

### Non-communicable Disease Service Readiness, Availability and Utilization

Our study is the first multi-site study comparing NCD service readiness, availability and utilization around prevention, screening, and treatment at the CHC level in Vietnam. This is also the first study to examine NCD services in a mountainous region that consists predominately of ethnic minorities. Our findings suggest that NCD service readiness, availability and utilization varies among the different disease category with hypertension and mental illness being most available and CLDs, diabetes and cancer being least available. The inter-province variability is significant, with services in the mountainous region being least available for all NCDs except mental illness.

It is notable that mental illness (schizophrenia and epilepsy) services are widely available in all 3 study provinces. The NTP on community mental health was established in 1999, and became a part of the National NCD Program in 2002 as the National Mental Health Program (NMHP).^15,19^ The goal of the program is for community management of schizophrenia and epilepsy.^15,19^ This program is funded by the central and provincial governments, and implemented through a network of national and provincial psychiatric hospitals, which was established in 1999.^15^ Patients are referred from the CHC to provincial hospitals for treatment and establishment of medication regimens.^15^ Following stabilization, patients return to their CHC for continued management.^15^ This program also has a strong community awareness promotion component which utilizes television, magazines and other forms of mass media.^15^

Regarding NCD readiness, all CHCs in our study met national facility standards outlined in Ministry of Health Decision 3447/QĐ-BYT (September 22, 2011) for staffing, infrastructure and equipment availability for NCD screening activities,^34^ which supports the higher proportion of reported services around NCD screening as compared to treatment. Despite expected capacity to deliver WHO’s and Vietnam’s package of essential medications for NCDs,^32,33^ our study found a limited number of medications available for NCD, except mental health. Access to multi-drug therapy for people with high risk of developing myocardial infarction and stroke are considered evidence-based “best buy” interventions that are not only highly cost-effective but also feasible and appropriate to implement within the constraints of the local LMIC health systems.^35^ The limited supply of NCD medications at CHCs in our study may represent a major barrier to NCD service availability. Medications are obtained from the district hospital on a monthly procurement schedule, often based on previous month’s supply and demand. Our study found less than 15% of total visits to CHCs were NCD related. Low service utilization for NCDs may contribute to the low supply of NCD medications available at the CHC, resulting in a negative feedback loop.

While NCD financing was not a focus of this study, it may contribute to this negative feedback loop. The majority of CHCs in Vietnam are not contracted with the health insurance system. Therefore clinical encounters, medications, and diagnostic services at CHCs are out-of-pocket expenses.^36^ Lower utilization rates for NCD services may contribute to hospital overcrowding as patients skip the CHC and directly self-refer to the hospitals for examination and treatment.^7^ The rate of self-referrals is approximately 42% in provincial hospitals, 59% in central hospitals and 93.5% at the specialist hospitals/institutes.^7^

An additional factor that may influence the limited NCD service availability is the capacity and knowledge of CHC staff to address NCDs. Thi et al studied all CHCs in one district and concluded that healthcare workers were unqualified to provide screening, diagnosis, and treatments of NCDs.^24^ Our study also found that CHC utilization was statistically significantly different among the study provinces, with the total mean number of visits lower in the mountainous region. However, because we do not have the total number of people in each CHC, we cannot make a meaningful comparison of clinical utilization rates. Our findings were consistent with previous studies which demonstrated that ethnic minorities have lower clinical utilization rates possibly due to government policies and programs which appear lacking in appropriate cultural adaptation and sensitivity.^21-23^ Additionally, there have been reported examples of discrimination from health staff toward ethnic minority persons.^23^ Finally, cultural norms among some ethnic minority groups to utilize traditional healers and shamans may contribute to lower CHC utilization rates.^23^

### Non-communicable Disease Prevention

Our study examined the 4 modifiable risk factors that NCDs share (tobacco consumption, alcohol consumption, physical inactivity and unhealthy diets) and found that less than a quarter of CHCs conducted NCD prevention services, mostly in the form of mass communication through loud speaker broadcasts rather than individualized counselling. Although individual counselling is less effective than population-level interventions, when applied consistently, patient education may have considerable impact.^37^ Our study also found that CHCs in the mountainous region conducted prevention activities less often than as those in the other 2 regions, even though this region has a greater percentage of smokers and people reporting drinking 1-4 days/week, putting them at greater risk for developing NCDs.^31,35^

### Primary Care Strategy

Our study suggests the need for an integrated strategy to address the NCD epidemic at the CHC level. This integrated strategy may include an examination of the current policies and regulations around CHC organization and health insurance reimbursement. As Vietnam aims to achieve UHC, new, more proactive strategies to reimburse CHCs for NCD services may increase patient utilization. Additionally, CHCs may require a fundamental workflow re-design, enabling them to move away from sites that implement vertical NTPs into clinics that horizontally integrate programs for preventing, diagnosing, and managing diseases to promote the health of the population they serve.

There is support for primary care strengthening within the Communist Party of Vietnam and the government.^8,38-41^ Most recently, the government underscored the importance of NCD prevention and control with Prime Ministerial Decree 376 issued in 2014, creating a legal framework for creating systems for NCD prevention, treatment, and control at the community level.^41^ Resources have been committed to supporting primary care strengthening. The government launched a new US$1.2 million project in 2015, Health Professional Education and Training Project (HPET), which supports human resource development for primary care providers.^7^ However, additional research needs to be done at the primary care level to understand the current challenges and limitations, specifically around health information systems, health financing and health worker knowledge, to inform policies and programs that will contribute to the transformation of these CHCs.

## Conclusion

While NCD services for prevention, treatment and management are not readily available at the CHC level in northern Vietnam, services for schizophrenia and epilepsy are widely available. A number of factors may contribute to limited service availability, including that CHCs have historically served as implementation sites for national NTPs, often lack first line NCD medications, and have a low clinic utilization rate. The NCD epidemic may have a disproportionately negative impact on the mountainous region which has increased exposure to NCD risk factors and lower availability of NCD prevention, total mean clinic visits, and availability of NCD services.

The Vietnamese government has designated a strong health sector and expanding health coverage as national priorities in the socio-economic development of the country for 2015-2020. In 2009, the government began to implement UHC. To effectively respond to the increase coverage in health insurance, the government will need to make investments and efficiently utilize the limited resources for health. Transforming the under-performing CHCs to reflect the epidemiological burden of disease and increase service availability, readiness, and utilization for NCDs will be a crucial step towards creating a robust and responsive health system. Our multi-site study adds to the body of evidence that the CHC, in its current state, is not responsive to the NCD epidemic. The government and its international development partners will need to make significant and strategic investments to increase NCD service availability and clinic utilization.

## Study Limitations

This study is descriptive and hypothesis-driven. While, for the facility assessment, the study utilized the validated WHO SARA tool, we modified the tool for the Vietnam context and did not validate the final tool. In addition, the study only focused on northern Vietnam. Therefore, the results may not be generalizable to the rest of Vietnam, particularly to large urban areas. The study also relied predominately on self-reported questionnaires which may lead to both systematic and random errors. Finally, we did not assess provider knowledge and competency around NCD or financing and referral mechanisms, all major factors in delivering NCD services.

## Acknowledgements

This work was supported by the US State Department Fulbright Program and Harvard Medical School Scholars in Medicine Office. The opinions expressed in this paper are the authors’ own and do not reflect the view of the Fulbright Program, the Department of State, or the United States government. The funders did not have any role in the study design, data collection, data analysis, data interpretation, writing of the manuscript, or the decision to submit for publication.

## Ethical issues

The institutional review boards of Hanoi Medical University, Hanoi, Vietnam and Harvard Medical School, Boston, MA, USA approved this study.

## Competing interests

Authors declare that they have no competing interests.

## Authors’ contributions

DBD, ALE, HVM, and LHN provided input into the study design, data collection, and data interpretation. DBD drafted the manuscript with comments from ALE, HVM, and LHN. DBD had access to all of the data and final responsibility for the decision to submit for publication.

## Authors’ affiliations

^1^Department of Medicine, Brigham and Women’s Hospital, Center for Primary Care, Harvard Medical School, Boston, MA, USA. ^2^Hanoi University of Public Health, Hanoi, Vietnam. ^3^Department of Medicine, Beth Israel Deaconess Medical Center, Harvard Medical School, Boston, MA, USA. ^4^Division of Global Health Equity, Brigham and Women’s Hospital, Center for Primary Care, Harvard Medical School, Boston, MA, USA.

## Key messages

**Implications for policy makers**

After reviewing our findings, we hope that policy makers will:

- Appreciate that non-communicable disease (NCD) services for prevention, treatment, and management may not be readily available at the commune health center (CHC) level.
- Consider the need for an explicit focus on disadvantaged populations, including ethnic minorities, in national NCD policy and investments.
- Understand that investments may be needed to transform CHCs so they are prepared to address NCDs.
- Consider prioritizing investments in NCD awareness and prevention efforts at the community level.

**Implications for public**

Commune health centers (CHCs) in Vietnam have made significant strides in managing acute infectious diseases and implementing national disease-specific vertical programs. However, in the context of socioeconomic development, the burden of disease in Vietnam has transitioned such that there is a far greater prevalence of non-communicable diseases (NCDs), which require an integrated, longitudinal approach to care. This study identified that CHCs in Vietnam have limited capacity to prevent, diagnose, and treat NCDs in 3 provinces in northern Vietnam, especially among less wealthy and ethnic minority areas.
